# Technology and Reflection: Mood and Memory Mechanisms for Well-Being

**DOI:** 10.1186/s13612-016-0045-3

**Published:** 2016-06-15

**Authors:** Artie Konrad, Simon Tucker, John Crane, Steve Whittaker

**Affiliations:** University of California at Santa Cruz, 1156 High Street, Santa Cruz, CA USA; Google, 1600 Amphitheatre Pkwy, Mountain View, CA USA

**Keywords:** Memory, Mood, Emotion, Well-being, Reflection, Technology mediated reflection, Reminiscence

## Abstract

**Background:**

We report a psychologically motivated intervention to explore Technology Mediated Reflection (TMR), the process of systematically reviewing rich digital records of past personal experiences. Although TMR benefits well-being, and is increasingly being deployed, we know little about how one’s mood when using TMR influences these benefits. We use theories of memory and emotion-regulation to motivate hypotheses about the relationship between reflection, mood, and well-being when using technology. We test these hypotheses in a large-scale month long real world deployment using a web-based application, MoodAdaptor. MoodAdaptor prompted participants to reflect on positive or negative memories depending on current mood.

**Methods:**

We evaluated how mood and memory interact during written reflection and measured effects on well-being. We analyzed qualitative and quantitative data from 128 participants who generated 11157 mood evaluations, 5051 logfiles, 256 surveys, and 20 interviews.

**Results:**

TMR regulated emotion; when participants reflected on memories with valences opposite to their current mood, their mood became more neutral. However this did not impact overall well-being. Our findings also clarify underlying TMR mechanisms. Moods and memories competed with each other; when positive moods prevailed over negative memories, people demonstrated classic mechanisms shown in prior work to influence well-being. When negative moods prevailed over positive memories, memories became negatively tainted.

**Conclusions:**

Our results have implications for new well-being interventions and technologies that capitalize on the interconnectedness of memory and emotion.

## Background

Our emotions and memories are intertwined, each influencing the other. For example, *autobiographical memory*, the personal memories from our lifetime, plays a critical role in enhancing positivity (Conway and Pleydell-Pearce 2000). In turn, our current mood shapes memories by filtering what we remember (Berntsen 2002; Matt et al. 1992). *Mood*-*congruent memory*, for instance biases us to remember past experiences that are emotionally consistent with current mood. However, in other contexts, people remember *mood*-*incongruent* memories to regulate emotions, i.e., recalling positive past experiences to improve current negative moods (Erber and Erber 1994).

Past memories can also be actively reviewed, a process known as *reflection*. Reflection can also be done using technology, in *technology mediated reflection* (TMR). For instance, TMR tools such as Timehop, MorningPics, Pensieve, and Echo provide rich, detailed records of past personal memories in the form of videos, images and text to facilitate reflection. Like autobiographical memory, TMR enhances well-being, albeit through different mechanisms (Isaacs et al. 2013). However, while TMR is increasingly common, it is not well understood, and in particular it is unknown whether *mood* affects its benefits. We know that mood primes congruent, and in some contexts, incongruent memories to help modify behavior and emotions. But how does mood impact our ability to benefit from systematically reviewing the past in TMR? If we are in a positive rather than negative mood, is it easier to learn from and find the bright side of negative memories? Might we benefit more from revisiting enjoyable memories while in a negative mood when most needing mood enhancement? These questions have implications both for technology and for theories of well-being.

To answer these questions about relationships between mood, reflection and well-being, we developed and deployed a web-based TMR application, MoodAdaptor. Two versions of MoodAdaptor prompted participants to reflect on past memories that were the *opposite* of current mood (mood-incongruent), and two versions prompted reflection on memories *consistent* with current mood (mood-congruent). Comparing mood-incongruent with mood-congruent reflection for each memory valence (positive and negative) determined whether current mood influences self-reflection, what mechanisms are at play, and how this affects well-being.

### Autobiographical Memory Biases

Autobiographical memory encodes, stores and retrieves information about personal experiences. It has important self-enhancing biases that preserve well-being (D’Argembeau and Van der Linden 2008). People remember about twice as many positive as negative memories (Walker et al. 2003). The *fading affect bias* characterizes how emotions associated with negative memories fade faster than emotions associated with positive memories (Walker et al. 2003). Also, people have a “rosy view” of the past, remembering past memories more positively than their actual experience of the event, enhancing well-being (Mitchell et al. 1997).

### Emotion and Memory

While adaptive memory biases enhance our positivity, emotion in turn has a profound effect on our memories. There are two opposing mechanisms at work that are triggered in different situations. *Mood*-*congruent* memory primes memories whose emotional content matches current mood. A depressed mood primes access to negative memories like failures and disappointments The effect has been demonstrated in different settings, across a wide range of moods and for different types of memory (such as conscious explicit memories and unconsciously primed memories) (Bower 1981; Matt et al. 1992; Watkins et al. 1996). Recalling similarly valenced past experiences is argued to help guide behavior by providing past information relevant to the current situation (Bower 1981; Pillemer 1992; Levine and Pizarro 2004). For example, the fear induced by seeing a snake will prime access to prior fearful memories of snake experiences to help navigate the current situation. Similarly, in self-concordant theory, behaviors are more likely to be performed when they are consistent with one’s prior experiences and dispositions (i.e., needs, values, and motives) (Sheldon and Lyubomirsky 2007). Thus behaviors might also be more likely when memories are concordant with current mood, as in mood-congruent memory.

In other contexts, however, people selectively retrieve *mood*-*incongruent* memories to regulate mood (Parrott and Sabini 1990; Erber and Erber 1994; Rusting and DeHart 2000). People retrieve happier memories when in negative than positive moods (Parrott and Sabini 1990). Students are more likely to recall mood-incongruent memories before class to regulate emotions for more level-headedness around their peers (Erber and Erber 1994). And adaptive mood-incongruent strategies can be induced by providing people with specific instructions to engage in positive reappraisal—reinterpreting negative memories to extract positive outcomes (Rusting and DeHart 2000). Thus, while mood-congruent memory is well-documented, and may function to guide behavior, mood-incongruent strategies potentially aid emotion-regulation and well-being.

However, there could be negative consequences to mood-incongruent memory. For example, while positive memories help regulate negative mood, the negative mood in turn can taint the positive memory. This is called *kill*-*joy thinking,* which involves re-assessing positive memories to uncover overlooked negative aspects (McAdams et al. 2001; Bryant and Veroff 2007; Quoidbach et al. 2010). Kill-joy thinking correlates with reductions in well-being (Schwartz et al. 2002; Larsen and McKibban 2008; Polman 2010).

### Unmediated Reflection

Unmediated reflection involves mentally reviewing our memories of past experiences without employing technology. Unmediated reflection benefits both physical and psychological health. Reflecting on positive memories (e.g., thinking about past successes, friendships) is adaptive and increases perceived enjoyment of life (Bryant et al. 2005). Positive reflection also increases positive affect and is often invoked to cope with painful affective states like loneliness (Wildschut et al. 2006).

Counter-intuitively, reflecting on negative memories (which we call negative reflection) can promote general well-being. The *emotional writing* paradigm (Pennebaker and Beall 1986) explores the effects of negative reflection, by having participants repeatedly write about past traumas with consistently demonstrated well-being benefits (Smyth 1998). However, these benefits occur over the long-term; emotional writing induces an immediate negative reaction, although this reduces over time and repeated writing sessions (Sloan and Marx 2004).

The exact *mechanism* for the success of emotional writing remains unclear. Different theories suggest that emotional writing is effective because of increased *understanding, redemption* and *distancing.*

#### Understanding

Emotional writing helps structure traumatic experiences into a coherent narrative (Pennebaker et al. 1997; Pennebaker and Chung 2007). Structuring allows painful experiences to be better understood, reducing their emotional intensity and improving well-being (Smyth et al. 2001). Increased understanding is signaled by words that indicate insight (‘think’, ‘know’, ‘consider’) and causal reasoning (‘because’, ‘reason’, ‘hence’) which increase across writing sessions.

#### Redemption

By contrasting current and past feelings about negative situations, people see that they overcame difficult experiences. This contrast constitutes a redemption sequence—shifting a previously negative perception of an experience to a more positive, triumphant evaluation (McAdams et al. 2001). Again this increases well-being (Wildschut et al. 2006).

#### Distancing

Over time, people’s writing about past negative experiences becomes less self-focused, demonstrating adaptive distancing (Campbell and Pennebaker 2003; Rude et al. 2004). When people experience emotional or physical pain, attention is self-focused, as reflected in their language use. For example, depressives use more first-person pronouns (Rude et al. 2004; Niederhoffer and Pennebaker 2002). Furthermore, shifting from first- to third-person event descriptions over time promotes health improvements (Campbell and Pennebaker 2003).

### Technology Mediated Reflection

Similar to unmediated reflection, TMR systems facilitate remembering autobiographical memories, but such systems also enhance this process by capturing rich records in the form of images, videos or textual descriptions of past personal experiences. These detailed records potentially allow more accurate and comprehensive reflection. Systems such as Timehop, Askt, Echo, My Wonderful Days, Live Happy, MorningPics, and 1 Second Everyday send back past records, allowing systematic reflection on recorded experiences after time has passed. For example, users might be presented with photos they took or posts they made and asked to reflect on how they now feel about those past events. Other tools such as PosiPost Me, Moodmill, MobiMood, and eMoto support social reflection by sharing emotional data. Facebook has explored TMR on past posts with On This Day, Year in Review, Timeline Moviemaker, Lookback videos and Say Thanks. These systems are becoming more common, for example Facebook’s Lookback videos has been accessed by over 200 million people (Bandaru 2014). Recently, technology companies have also examined users’ moods in large-scale studies (Fowler and Christakis 2008; Rosenquist et al. 2011; Kramer et al. 2014). However, to our knowledge, no prior work explores relations between mood and reflection, nor underlying mechanisms, the primary objective of this paper. There are important practical implications too; if mood does influence TMR, millions of people might not be optimizing their well-being benefits, and we need to better understand technologies that are so widely deployed.

One of the best studied TMR systems is Pensieve. It provides users with past Facebook status posts, asking them to write current reactions to those memories (Peesapati et al. 2010). Participants reported enjoying this reflective process and that it improved mood. Echo (Isaacs et al. 2013) facilitated recording of experiences as they happened (rather than retrospectively providing past Facebook posts as with Pensieve). The researchers found that capturing three experiences per day for one month, and reflecting on these experiences by writing about them, increased well-being. Echo also supported other classic reflection benefits (Isaacs et al. 2013). For example, redemption sequences were identified through increased positive affect words (e.g., ‘love’, ‘nice’) and words indicating acceptance (e.g., ‘ok’, ‘agree’) used by participants when reflecting on past negative experiences. And understanding was expressed via insight words (e.g., ‘think’, ‘know’, ‘consider’) and words signaling cognitive processing (e.g., ‘cause’, ‘know’, ‘ought’).

### Research Questions

Both TMR and unmediated reflection improve well-being. However, prior work has not addressed whether well-being benefits are influenced by *current mood*, and it has not explored this in the context of TMR. We know that mood triggers memories in opposing ways, where memory can either be mood-congruent (Bower 1981), or mood-incongruent to regulate current mood (Parrott and Sabini 1990; Erber and Erber 1994; Rusting and DeHart 2000). However, little is known about how mood and reflection interact in TMR. Our research questions therefore include: Does people’s current mood influence the well-being benefits they derive from reflecting on their pasts? Additionally, does reflection valence influence one’s current mood?

Our goal was to understand how mood and reflection interact in TMR by comparing congruent with incongruent reflection strategies. However, because positive and negative memories are so different in the ways they influence well-being, we formulated different hypotheses for each. We predicted that negative reflection when in a positive mood would increase general well-being more than negative reflection when in a negative mood. These well-being increases should be driven by three mechanisms: understanding, distancing, and redemption. Negative reflection when in a positive mood introduces a different emotional state from the initial experience, which should encourage new perspectives and understanding (Petrie et al. 1998; Boals and Klein 2005; Pennebaker and Chung 2007). Also, positive moods might encourage less self-focus than negative moods, allowing for adaptive distancing from negative memories (Campbell and Pennebaker 2003; Rude et al. 2004). Lastly, contrasting one’s current positive emotional state with negative feelings about a past negative experience, may help people see they overcame the difficult experience, contributing to redemption sequences (Rusting and DeHart 2000; Wildschut et al. 2006). However, we also predicted a side-effect to these adaptive mechanisms, namely that negative reflection would contaminate one’s currently positive mood (Sloan and Marx 2004). Thus, we expected general well-being benefits [consistent with Pennebaker and Beall (1986)] due to increased distancing, redemption and understanding, but short-term costs to one’s currently positive mood [consistent with the negative short-term responses demonstrated by Sloan and Marx (2004)].

We also predicted that positive reflection when in a negative mood would have greater momentary benefits than when in a positive mood. Positive reflection is often invoked naturally as an emotion-regulation strategy to reduce negative affect (Erber and Erber 1994; Bryant et al. 2005; Wildschut et al. 2006). In contrast, if already in a positive mood, there may be reduced room for improvement, experiencing a ceiling effect for positive reflection.

However, incongruent positive reflection may have undesirable side-effects where the memory itself becomes tainted with newly identified negative details (Bryant and Veroff 2007). While improving our emotional stance on past traumas may genuinely benefit long-term well-being, positive reflection while in a negative mood may have the opposite effect through maladaptive kill-joy thinking. Thus we predicted that positive reflection when in a negative mood would have momentary mood benefits, but general negative well-being consequences.

While it might be possible to test some of these effects short-term in the lab, we felt it was important in assessing effects of TMR on well-being that we examined real-world behaviors over a longer period. We therefore ran a month-long real-world reflection intervention. To summarize, here are our four hypotheses. They explore relations between mood, reflection and well-being in TMR, as well as underlying mechanisms:

Negative reflection when in a positive mood (incongruently) versus negative mood (congruently) will:

#### Hypothesis 1

Reduce momentary mood.

#### Hypothesis 2

Increase general well-being, through greater distancing, understanding, and redemption.

Positive reflection when in a negative mood (incongruently) versus positive mood (congruently) will:

#### Hypothesis 3

Increase momentary mood.

#### Hypothesis 4

Reduce general well-being, through greater kill-joy thinking.

To evaluate our hypotheses, we developed MoodAdaptor, a web-based application accessible from any smartphone browser. MoodAdaptor elicits written reflection based on current mood. Four different versions of MoodAdaptor were deployed to four experimental groups: an Incongruent Positive version, Congruent Positive, Incongruent Negative, and Congruent Negative. To help clarify the condition names used throughout this paper: the second word in the condition refers to the valence of memory reflected on (i.e., Incongruent Negative means people reflected on negative memories). Thus we were able to compare the benefits derived from mood-incongruent reflection against mood-congruent reflection for each emotional valence.

## Method

### Participants

One hundred and thirty one participants were recruited through Facebook, university email lists and official Facebook groups, using a snowball recruiting strategy where participants could recruit others. They were paid $50 for completing the study and an additional $30 for each person they recruited who also completed the study. Participants were randomly assigned to a group, roughly balanced across gender and age, and not informed there were different groups. Three dropped out because of unexpected family emergencies, and becoming too busy to continue. This left 128 participants who completed the study (91 female), aged 18–62 (M = 24.56, SD = 8.87). There were 34 in the Incongruent Negative (23 female, age M = 23.94, SD = 7.70), 34 in the Congruent Negative (25 female, age M = 24.85, SD = 9.06), 30 in the Incongruent Positive (22 female, age M = 25.60, SD = 11.58), and 30 in the Congruent Positive group (21 female, age M = 23.90, SD = 6.90).

### Materials

Participants’ well-being was assessed using three validated standard scales at pretest and posttest. These are the Subjective Happiness Scale, Satisfaction With Life Scale, and Ryff Scales of Psychological Well-Being. Because there is no universal measure of well-being, we included both hedonic (e.g., pleasure, satisfaction) and eudaimonic (e.g., meaning, personal growth) scales to triangulate different measurement perspectives (Deci and Ryan 2008). We did not have differing predictions for each scale, but instead expected all three scales to increase for Hypothesis 2, and decrease for Hypothesis 4. This is because positive and negative memories have hedonic qualities of pleasure and unpleasantness, but also may encourage eudaimonic benefits from increased self-understanding and personal growth upon reflection.

#### Subjective Happiness Scale (SHS)

The SHS consists of four items to assess global subjective happiness using absolute ratings and ratings of self relative to perception of others (Lyubomirsky and Lepper 1999). The SHS has high internal consistency, reliability, and convergent and discriminant validity (Lyubomirsky and Lepper 1999). An example item is, “Compared to most of my peers, I consider myself…” with response categories ranging from “less happy” to “more happy.”

#### Satisfaction with Life Scale (SWLS)

The SWLS consists of five items to assess satisfaction with life as a whole (Diener et al. 1985). It does not query specific life domains but instead allows participants to weigh these domains overall. The SWLS shows strong internal reliability, test–retest stability, and convergent and discriminant validity (Diener et al. 1985). An example item is, “If I could live my life over, I would change almost nothing,” with response categories ranging from “strongly disagree” to “strongly agree.”

#### Ryff’s Scales of Psychological Well-Being (RPWB)

The RPWB is a theoretically grounded instrument reflecting six facets of eudaimonic well-being: autonomy, environmental mastery, personal growth, positive relation with others, purpose in life, and self-acceptance. The original RPWB consisted of 120 questions (Ryff 1989). We used the 54 item version to reduce participant burden taking the surveys, and because this version is being used in large ongoing studies (Hauser et al. 1992). The RPWB shows high internal consistency, test–retest reliability as well as convergent and discriminant validity, and accounts for additional variance beyond that covered by hedonic measures (e.g., life satisfaction, happiness) (Hauser et al. 1992; Ryff 1989). An example item is, “When I look at the story of my life, I am pleased with how things have turned out,” with response categories ranging from “strongly disagree” to “strongly agree.”

### The MoodAdaptor System

MoodAdaptor was designed specifically for this study and has not been employed in prior work. MoodAdaptor prompted participants to write about their memories, asked participants to rate their mood three times per day, and then sent back specific memories for written reflection depending on their mood state and experimental condition. We now discuss a pilot of these procedures followed by a description of each.

#### Lab-Based Pilot Study

All aspects of the procedure (number of memories elicited, orienting instructions, intervention duration, emotion rating method) were developed after extensive piloting with 30 participants not included in the main intervention. All 30 participants were students at UC Santa Cruz and provided with course credit for participating. We asked ten students to write out ten memories of each valence, a different ten students wrote 15 memories of each valence, and a final ten wrote 20 memories of each valence. We interviewed everyone about the clarity of our instructions, and how challenging it was to generate memories. Most people said they were comfortable generating up to 15 memories of each valence. Students returned to the lab a month later. We had them rate their current mood, reflect on one of the memories they previously wrote, rate their mood a second time, then we interviewed them about the experience. The following instructions and procedures are the result of this pilot and student interviews.

#### Pool of Memories

Participants first logged-into MoodAdaptor on a computer and were asked to write about 15 positive memories and 15 negative memories. Participants received the following instructions:

“Now we’d like you to write about some of your memories. Please describe in detail 15 recent events or experiences that made you feel good when they occurred, and 15 that made you feel bad when they occurred. These events should be within the past year, and the more recent and emotional the better. Also, please favor events that you consider open. Open events have current relevance and are unresolved.”

We imposed a minimum length requirement of 90 characters to ensure each memory had sufficient detail for when people later reflected about it. Next, participants generated an emotional rating of the memory:

“How much positive or negative emotion did you experience at this event’s occurrence?” [with response categories on a 9 point scale ranging from “extremely negative” (1) to “extremely positive” (9) and a neutral response of “neither negative nor positive” (5)].

The majority of memories that were generated were clearly positive or negative, with few being ambiguous or including mixed emotions. This was also evident in the extremity of memory emotion ratings, with an average negative memory rating of 2.6, and average positive memory rating of 7.6. Here is an example of a typical positive memory: “My boyfriend’s sister just had a baby and they are already calling me Aunty. I am very excited to have this little one in the world and can’t wait to spend time with her!” And an exemplary negative memory is: “Walking in on my ex-boyfriend cheating on me and him doing nothing and not chasing me or trying to contact me after I fled the room was the worst pain I’ve felt in my life.” For reflections, we told participants that they should rate the strongest emotion they felt in cases where there were mixed emotions. Having a single judgment of emotion for memories and reflections was preferable to a complex series of evaluations of multiple facets such as the emotion circumplex that requires training for reliable deployment (Scherer 2005; Ghallab 2008).

#### Personal Emotion Scale

Participants rated their emotions at multiple points in the study. To encourage consistency in emotion ratings, we used a method deployed in a similar study (Isaacs et al. 2013). When participants first logged-into MoodAdaptor, they were prompted to create a personal emotion scale (1–9) to consistently calibrate emotional reactions. We asked them to assign an actual experience to each number on the scale. On completion, the results were saved and accessible via a hyperlink, allowing access to the scale throughout the study. We encouraged participants to continue consulting this scale in weekly check-in interviews.

#### Mood Probe and Reflection

**Fig. 1:**
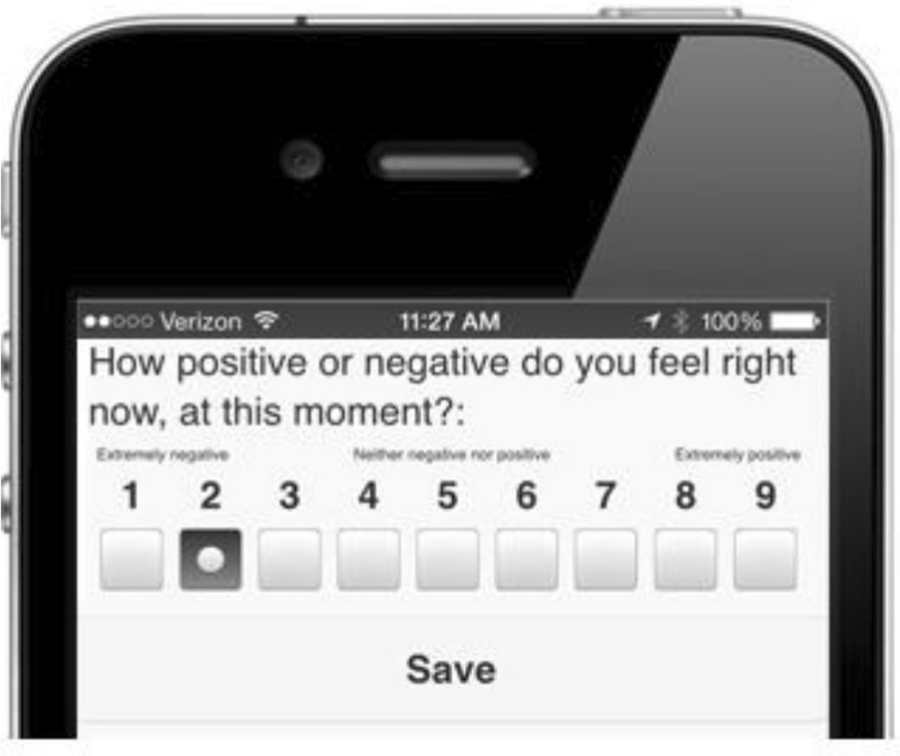
Interface of a mood probe in MoodAdaptor

For the duration of the study (30 days), participants received three daily mood probes at random times between 10 a.m. and 9 p.m. This procedure of thrice daily probes is common in experience sampling studies (LeFevre et al. 1985). These probes arrived via a link sent by text messaging to the participant’s phone. If a participant didn’t respond within an hour, they received a follow-up text reminder. Clicking on the link took participants to MoodAdaptor online and the following mood probe (see Fig. 1):

“How positive or negative do you feel right now, at this moment?” (with response categories on a 9 point scale ranging from “extremely negative” to “extremely positive” and a neutral response of “neither negative nor positive”).

How participants responded to this probe (positively or negatively) determined which memory they received for reflection. However, even if they were eligible for receiving a memory, they didn’t always get one. There were fewer memories in the pool than mood probes, requiring that we ration memories so participants wouldn’t use them up prematurely. However, if a memory was selected for reflection, it was presented directly after the mood probe to allow the participant to read the memory description they had previously written, along with its accompanying emotion rating. Below this description and emotion rating were instructions to reflect on the memory by re-writing about it (again with a 90 character limit):

“After reading and thinking about the above reflection, please write 2–3 sentences about your current feelings regarding the event.” They were next given another 9 point emotion scale and asked to:

“Rate how positive or negative you *now* feel about the event.” Reflecting on a memory removed it from the pool. Lastly, after reflecting, participants were given a second mood probe to assess momentary changes in mood due to reflection.

#### System Version 1—Incongruent Positive Group

When this group responded *negatively* to the mood probe (i.e., choosing a 1–4 on the emotion scale), they sometimes received a *positive* memory for reflection. A positive memory was defined as any memory in the pool with an emotion rating of six or above. So, if this group responded negatively to the mood probe, they might next see a description of a positive memory they had generated, along with the emotion rating they assigned it. Below the description was an opportunity to reflect by re-writing about it. Whenever participants using this version rated their mood as neutral or above (five or above), they received nothing back (no system behavior).

#### System Version 2—Congruent Positive Group

This was similar to version 1, except when participants responded to the mood probe *positively* (six or above), they sometimes received a *positive* memory (six or above). They received no memories if they responded negatively or neutrally to the probe.

#### System Version 3—Incongruent Negative Group

When this group responded to the mood probe *positively* (six or above), they sometimes received a *negative* memory (four or below). They received no memories if they responded negatively or neutrally to the probe.

#### System Version 4—Congruent Negative Group

When this group responded to the mood probe *negatively* (four or below), they sometimes received a *negative* memory (four or below). They received no memories if they responded positively or neutrally to the probe.

#### Balancing Reflections Across Groups

The goal was to roughly balance reflections across the four groups. However, the negative mood groups (Incongruent Negative and Congruent Positive) had less opportunity for reflections because people are more often in a positive than negative mood. For example, our pilot study found that 60 % of mood probes were positive, 13 % were negative, and 27 % were neutral. To balance number of reflections, we made it more likely that negative mood groups received a reflection when they responded negatively to the mood probe.

### Procedure

**Fig. 2:**
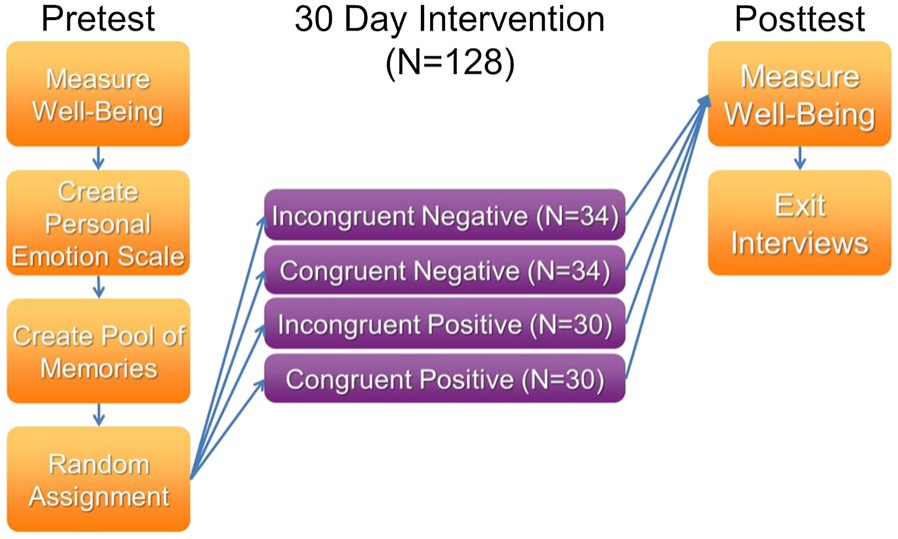
Flow chart illustrating the study’s procedures and sample sizes

The experiment was a randomized pretest–posttest field study with group (Incongruent Positive, Congruent Positive, Incongruent Negative, Congruent Negative) as manipulation, and three validated measures as dependent variables (see “Materials” section). See Fig. 2 for a flow chart illustrating the procedure. The study received ethics approval from UC Santa Cruz’s institutional review board. Participants completed the pretest survey online, and the same survey at posttest after working with MoodAdaptor for 30 days.

After completing the pretest survey, participants logged-into MoodAdaptor on their computer, and created their personal emotion scale. They then generated 15 positive, and 15 negative memories. This triggered the intervention, where MoodAdaptor probed mood three times a day, providing memories depending on the group the participant was assigned to. We called participants weekly to check-in and encourage compliance. After the intervention, participants took the posttest survey. In the final check-in phone call, we interviewed a subset of participants about their experiences.

## Results

### Hypothesis 1

Negative reflection when in a positive versus negative mood will reduce momentary mood

**Fig. 3:**
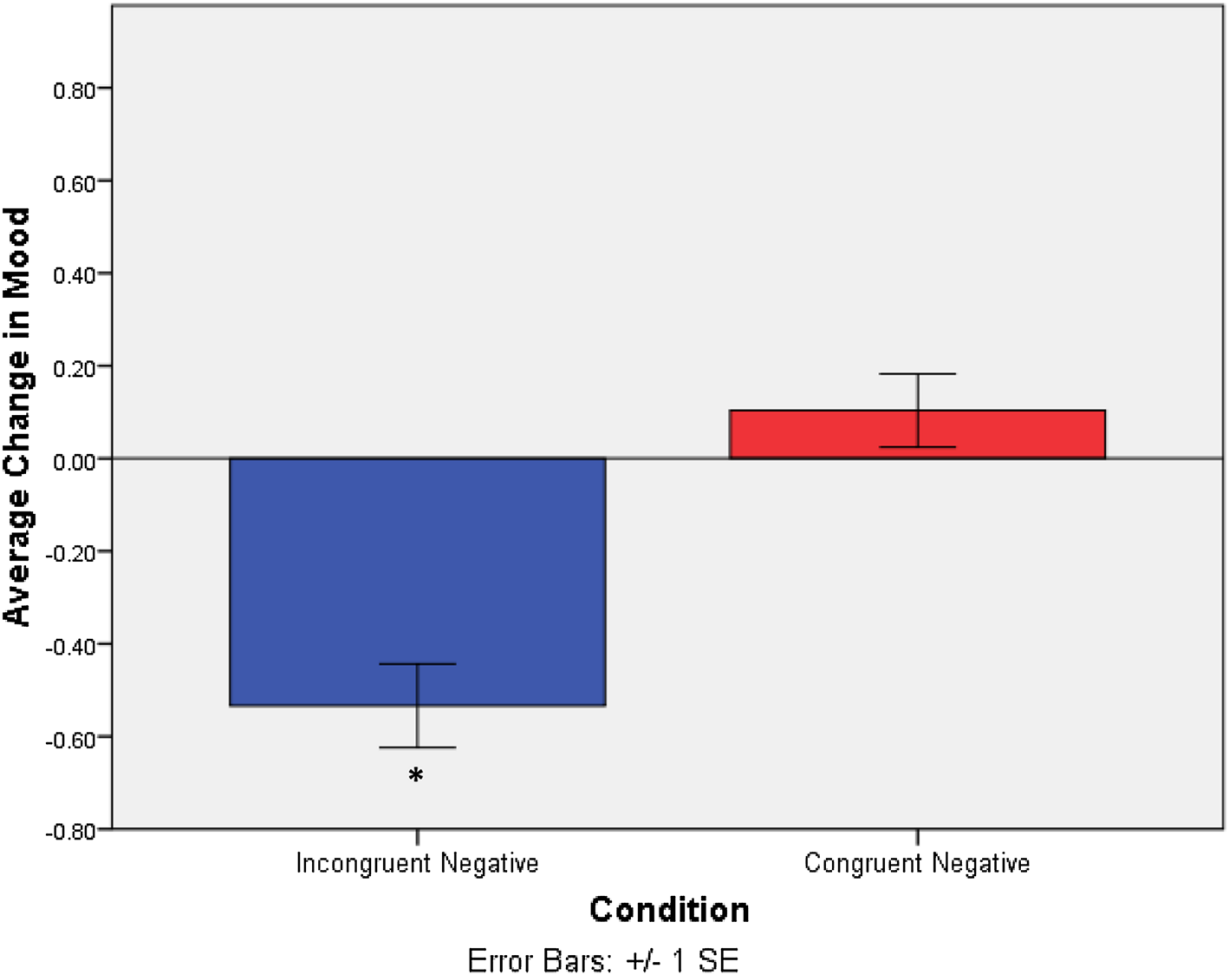
Average change in mood between pre versus post reflection mood probes for Incongruent Negative versus Congruent Negative groups. Shows a significant reduction in mood after reflection for the Incongruent Negative group. Note: significant findings are denoted by *asterisk*

For each reflection, there was a pre- and post-reflection mood probe. The difference between these scores was the change in mood, likely due to reflection. There was a high degree of compliance with responding to mood probes; participants completed on average 87.16 (SD = 4.98) of 90 possible initial mood probes. To assess Hypothesis 1, we ran an independent samples t test comparing groups (Incongruent Negative vs. Congruent Negative) on the average change in mood rating (with negative scores representing a detriment to momentary mood). Negative reflection when in a positive versus negative mood affected mood differently, *t*(62) = −5.24, *p* < .001, *d* = .89 (Incongruent Negative: MΔ = −.53, SD = .53, Congruent Negative: MΔ = .10, SD = .43). A one-sample t test comparing average change in mood to 0 revealed that the reduction in mood experienced by the Incongruent Negative group was significant with a large effect size, t(33) = −5.90, p < .001, d = 1.01 (see Fig. 3). In contrast there were no significant mood changes in the Congruent Negative group, t(29) = 1.31, p = .20. Hypothesis 1 was supported. In other words, reflecting on negative memories when in a positive mood reduces mood after reflection, whereas those in a negative mood showed unchanged mood.

We saw this contrast between Incongruent Negative and Congruent Negative in participants’ logfiles of memories and reflections. For example, this participant was in a positive mood, but negative reflection resurfaced feelings of anger:

### Incongruent Negative—Initial Memory

*That bitch. She has no right to demand money from us when she hasn’t done anything for this house. Does she not understand sunk costs?*

### Incongruent Negative—Reflection

*I really dislike her. So much. She was a terrible house mate and a terrible person****and it makes me angry just thinking about her***.

Whereas this participant who was already in a negative mood realized that negative reflection had no impact on her mood:

### Congruent Negative Initial Memory

*On the way back from San Diego, there was some serious traffic on the road. When we were driving past the accident, I saw a body on the floor covered with a sheet…*

### Congruent Negative Reflection

*That’s still very sad, but I am preoccupied with the drama between [L] and I right now.****I don’t think reflecting on this would make my emotions worse than they already are***.

### Hypothesis 2

Negative reflection when in a positive versus negative mood will increase general well-being, through greater distancing, understanding, and redemption

We assessed this hypothesis first by measuring general well-being changes, then explored the predicted mechanisms by analyzing the content of memories and reflections. Survey data was analyzed using a mixed-design multivariate analysis of variance (MANOVA) with one between factor (Incongruent Negative vs. Congruent Negative) and one within factor (pretest vs. posttest). The dependent variables were the three well-being scales (SHS, SWLS and RPWB). See Table 1 for means and standard deviations for the well-being scales by group at pretest and posttest. The MANOVA results showed no significant main effects for time (*V* = .03, *F*(3,64) = .54, *p* = .66) or condition (*V* = .02, *F*(3,64) = .41, *p* = .74), and no significant interaction (*V* = .02, *F*(3,64) = .47, *p* = .71). Because these overall effects were not significant, we did not follow-up with univariate ANOVAs or specific subscales of the RPWB.

**Table 1:** Means and standard deviations for three survey measures of well-being for Incongruent Negative versus Congruent Negative at pretest and posttest

Well-being survey	Incongruent negative (n = 34)		Congruent negative (n = 34)	
	Pretest	Posttest	Pretest	Posttest
SHS				
Mean	75.29	75.29	70.86	71.86
Std Dev	17.71	17.00	17.57	14.00
SWLS				
Mean	71.6	71.09	69.31	68.40
Std Dev	17.33	17.23	18.86	19.34
RPWB				
Mean	76.97	75.79	74.96	74.94
Std Dev	11.43	11.38	10.42	9.94

All scores are normalized to a 100 point scale

However, some participants were rarely in a negative mood, reducing the average number of reflections completed for the Congruent Negative group, but increasing it for the Incongruent Negative group (Incongruent Negative: M = 12.62, SD = 2.43, Congruent Negative: M = 6.97, SD = 4.48). We therefore examined whether overall differences in number of reflections affected results. To provide greater homogeneity in number of reflections, we excluded Congruent Negative participants who completed fewer reflections than one standard deviation from the mean. Incongruent Negative participants who completed more reflections than one standard deviation from the mean were also removed. An independent t test comparing Incongruent Negative to Congruent Negative on number of reflections showed that there were no differences in number of reflections after these participants were removed, t(40) = 1.62, p = .11 (Incongruent Negative: M = 11.48, SD = 2.15, Congruent Negative: M = 10.26, SD = 2.73). As above, we ran a MANOVA on the change in survey scores after removing the atypical reflectors. We again found no significant main effects or interactions.

To investigate underlying mechanisms, for each memory and reflection, we used Linguistic Inquiry Word Count (LIWC) to analyze words that related to known well-being mechanisms. LIWC is a widely used linguistic analysis tool that calculates percentages of words used in different linguistic categories (Pennebaker et al. 2007). It has good internal reliability and external validity (as compared with human judges) (Pennebaker and Francis 1996; Kahn et al. 2007; Pennebaker et al. 2007; Tausczik and Pennebaker 2010). Although the LIWC dictionaries are able to measure up to 72 different linguistic categories, we focused here only on categories that directly concerned our hypotheses and that have been demonstrated to relate to emotional well-being in previous reflection studies. Specifically, we targeted word categories that provided evidence of distancing, understanding, and redemption. Distancing was measured through usage of personal pronouns (‘I’, ‘you’, ‘we’) and tense (past, present, future) (Campbell and Pennebaker 2003). Understanding was measured through usage of insight words (‘think’, ‘know’, ‘consider’) and cognitive processes (‘cause’, ‘know’, ‘ought’) (Petrie et al. 1998; Klein and Boals 2001). And redemption was measured through usage of affect words (‘happy’, ‘joy’, ‘love’) and indicators of acceptance (‘ok’, ‘yes’, ‘agree’) (Isaacs et al. 2013).

First we compared the Incongruent Negative and Congruent Negative groups for differences in words used in their reflections. The Incongruent Negative group used a greater percentage of words indicating redemption such as acceptance words, *t*(49) = 2.03, *p* = .048, *d* = .50, using Levene’s correction for heterogeneity of variance (Incongruent Negative: M = .19, SD = .30, Congruent Negative: M = .07, SD = .15). (Note that we used Levene’s correction for all subsequent t-tests with unequal variances). The Incongruent Negative group also used a greater percentage of words indicating distancing such as third-person plural pronouns, *t*(52.24) = 3.01, *p* = .004, *d* = .74 (Incongruent Negative: M = .73, SD = .71, Congruent Negative: M = .30, SD = .39). In contrast, the Congruent Negative group used a greater percentage of words demonstrating an inability to distance such as first-person plural pronouns, *t*(43.05) = −2.07, *p* = .04, *d* = .53 (Incongruent Negative: M = .43, SD = .56, Congruent Negative: M = .88, SD = 1.05). We also computed the change scores in the percentage of words used between the initial memory and its reflection. An independent t test of change scores again revealed greater percentage increases in acceptance words for the Incongruent Negative group, *t*(62) = 2.08, *p* = .04, *d* = .53 (Incongruent Negative: MΔ = .12, SD = .27, Congruent Negative: MΔ = −.01, SD = .22).

In the exit interviews, Incongruent Negative participants described experiencing redemption and distancing. For example, this participant discussed a key feature of redemption in seeing that she triumphed over past negative experiences:

### Incongruent Negative Exit Interview

*I was able to look back at some of the memories and see****that I’ve gotten past it***.

And this participant discussed distancing from negative memories as a result of his positive moods:

### Incongruent Negative Exit Interview

*I think that when I’m in a more positive state****I tend to distance myself from negative things****in order to stay in a positive state.*

Lastly, we examined how our target word categories used in reflections correlated with responses to specific well-being scales. For the Incongruent Negative group, reductions in Satisfaction with Life were correlated with a greater percentage of words that convey negative emotion (‘hurt’, ‘ugly’, ‘nasty’), *r*(32) = −.38, *p* = .03. In contrast for the Congruent Negative group, increases in Satisfaction with Life correlated with percentage of past tense words, *r*(28) = .38, *p* = .04. Overall, although our well-being predictions for Hypothesis 2 were not supported, there was evidence of two predicted mechanisms (redemption and distancing).

### Hypothesis 3

Positive reflection when in a negative versus positive mood will increase momentary mood

**Fig. 4:**
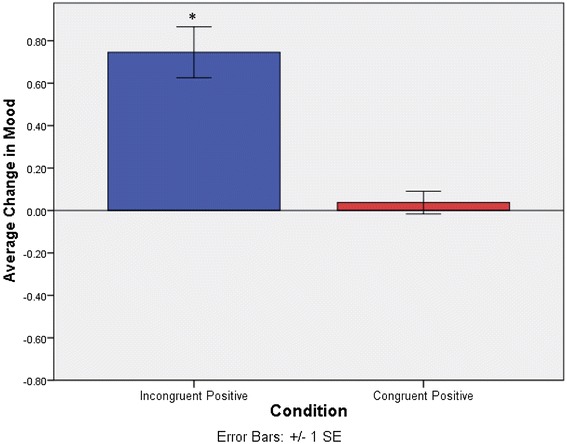
Average change in mood between pre versus post reflection mood probes for Incongruent Positive versus Congruent Positive groups. Shows a significant increase in mood after reflection for the Incongruent Positive group. Note: significant findings are denoted by *asterisk*

Now we turn to an investigation of positive memories. Hypothesis 3 was tested in the same manner as Hypothesis 1. We ran an independent samples t test comparing groups (Incongruent Positive vs. Congruent Positive) on the average change in mood scores (with negative scores representing a detriment to momentary mood). Positive reflection when in a negative versus positive mood affected mood differently, *t*(38.7) = −5.39*, p* < .001 (Incongruent Positive: MΔ = .75, SD = .65, Congruent Positive: MΔ = .04, SD = .29). A one-sample t test comparing average change in mood to 0 for each group revealed that the improvement in mood experienced by the Incongruent Positive group was significant with a large effect size, *t*(28) = 6.21, *p* < .001, *d* = 1.15 (see Fig. 4). In contrast there were no significant mood changes in the Congruent Positive group, *t*(29) = .70, *p* = .49. Hypothesis 3 was supported. In other words people who reflected on positive memories when in a negative mood experienced improvements in mood after reflection, whereas those in a positive mood showed unchanged mood.

We see this contrast in the exit interviews. For example, positive reflection when in a negative mood provided mood-elevation for this participant:

### Incongruent Positive Exit Interview

*I would be in a bad mood, I would be stressed at work, and you guys would surface something up that was really a bright spot for me and then instead of maybe a three,****I would bump up to a four****[on the emotion scale].*

Whereas this participant observed that positive reflection rarely had any impact on his positive moods:

### Congruent Positive Exit Interview

*From the prompt before the reflection and the prompt after the reflection…****there were very few times that was different***.

### Hypothesis 4

Positive reflection when in a negative versus positive mood will reduce general well-being, through greater kill-joy thinking

Survey data was again analyzed using a mixed-design multivariate analysis of variance (MANOVA) with one between factor (Incongruent Positive vs. Congruent Positive) and one within factor (pretest vs. posttest). The dependent variables were the three well-being scales (SHS, SWLS and RPWB). See Table 2 for the means and standard deviations for the well-being scales by group at pretest and posttest. The MANOVA results showed no significant main effects for time (*V* = .07, *F*(3,56) = 1.31, *p* = .28) or condition (*V* = .01, *F*(3,56) = .16, *p* = .93), and no significant interaction (*V* = .05, *F*(3,56) = .94, *p* = .43). Because these overall effects were not significant, we did not follow-up with univariate ANOVAs or specific subscales of the RPWB.

**Table 2:** Means and standard deviations for three survey measures of well-being for Incongruent Positive versus Congruent Positive at pretest and posttest

Well-being survey	Incongruent positive (*n* = 30)		Congruent positive (*n* = 30)	
	Pretest	Posttest	Pretest	Posttest
SHS				
Mean	73.14	74.57	77.43	75.14
Std Dev	14.86	13.71	14.71	14.29
SWLS				
Mean	68.29	70.86	70.00	72.86
Std Dev	17.71	17.46	17.63	14.69
RPWB				
Mean	75.26	76.09	77.13	77.04
Std Dev	9.04	10.79	9.92	8.92

All scores are normalized to a 100 point scale

Some participants were rarely in a negative mood, leading to group differences in the number of reflections completed (Incongruent Positive: M = 6.17, SD = 4.25, Congruent Positive: M = 11.87, SD = 2.98). As for Hypothesis 2, we removed atypical reflectors, and ran a follow-up MANOVA on the change in survey scores as above. We again found no significant main effects or interactions.

Once again, we used LIWC to dive deeper into mechanisms by examining word usage and well-being. First we compared group differences in words used in reflections. The Incongruent Positive group used a greater percentage of words conveying negative emotion than the Congruent Positive group, *t*(40.15) = 2.53, *p* = .02, *d* = .66 (Incongruent Positive: M = 1.90, SD = 1.42, Congruent Positive: M = 1.16, SD = .69). We also computed change scores in the percentage of words used in the initial memory compared with its reflection. An independent t test of these change scores revealed greater percentage increases in negative emotion words for those already in a negative mood, *t*(41.06) = 2.79, *p* = .01, *d* = .73 (Incongruent Positive: MΔ = 1.24, SD = 1.70, Congruent Positive: MΔ = .26, SD = .86). This is evidence of kill-joy thinking whereby positive memories become tainted due to one’s negative mood. We also examined how the language used in reflections correlated with responses to specific well-being scales. For the Incongruent Positive group, reductions in RPWB were correlated with greater percentage of past tense words, *r*(27) = −.45, *p* = .01. There were no significant correlations for the Congruent Negative group. Our well-being predictions for Hypothesis 4 were not supported, though there was evidence of the predicted kill-joy mechanisms.

In the exit interviews, Incongruent Positive participants described experiencing kill-joy thinking. For example, this participant described how negative moods influenced how he perceived positive memories:

### Incongruent Positive Exit Interview

*[There’s an] initial bias where if you’re feeling really low it’s hard to jump up and look at something with a clean slate or fresh eyes. So I think there’s some natural spill over there.*

## Discussion

We evaluated whether mood mediates the relationship between reflection and well-being, in a long-term real-world TMR study. We also examined how the memory valence during reflection influences one’s current mood state. We tested four hypotheses and the results inform research and practice in both mediated and unmediated reflection. As predicted, and consistent with mood incongruent memory, we found that in TMR, incongruent reflection is useful for mood-regulation. However, we found no evidence of changes in general well-being, although there were some specific effects. Our data also clarify mechanisms underlying mood-reflection relationships, by investigating the words used in memories and reflections.

Negative reflection when in a positive mood seemed to encourage redemption (acceptance words) and reduce self-focus (third-person plural pronouns) which may signal distancing from negative memories that involved other people. However, memories that resurfaced feelings of negativity (triggering negative emotion words in reflections) led to reductions in one well-being scale. In contrast, negative reflection when already in a negative mood seemed to discourage distancing (first-person plural pronouns), but this worked in participants’ favor, as remaining focused on the past (past tense usage) was associated with increases in one well-being scale. For positive memories, negative moods induced greater kill-joy thinking (negative emotion words) and reduced one well-being measure when reflections were focused on the past (past tense usage). This contrasts with negative memories, which seem to benefit from past-focused reflections when in a negative mood state, when there is no positivity to taint.

Negative reflection when in a positive mood reduced current mood, while positive reflection when in a negative mood enhanced it. This is consistent with prior work on unmediated memory showing that people sometimes select incongruent memories for mood-regulation (Erber and Erber 1994; Rusting and DeHart 2000). The current paper makes a contribution to memory and emotion literature by showing that the mood-regulating effects of incongruency can also be induced in mediated contexts, and identifying the mechanisms underlying these effects. But while incongruent mood-regulation seems to occur naturally in rather limited contexts, there may be strategic opportunities with technology to apply this technique more broadly. Technology might provide control over when and how mood is regulated in ways that regular memory doesn’t, i.e., people might receive targeted automatic prompts for positive reflection if they are in a negative mood.

This technique of structured incongruent reflection through TMR might benefit people seeking more equanimity in their moods. Always providing incongruent memories will move the extremes of mood closer to neutral, offering more balance. Mood-regulation of both positive and negative moods is an adaptive cognitive skill that has been explored in depth (Parrott 1993; Gross 1998; Larsen 2000). For example, a bearer of bad news may downgrade positive moods to be appropriate for delivering unwelcome news (Tesser et al. 1973). And conversely, if delivering positive news they may elevate their negative mood to show reciprocal happiness for the recipient. Technology mediated reflection may aid these scenarios through strategic incongruent mood-regulation.

Should elevating mood be preferred to equanimity, such as to ameliorate depression or dysphoria (Mitchell et al. 2010; Calvo and Peters 2014), positive memories could be reflected on when in a negative mood for mood-enhancement A couple of participants even mentioned this being useful for depression. For example, one participant told us: “A lot of the memories had a theme of hope in them. I suffer from mild depression in general in my life and I think that the big part of my depression is hopelessness, and so seeing the hope … that was the aspect that I would say brought me up the most.” Additionally, while mood-incongruent reflection on negative memories might be useful for mood-regulation, for mood-enhancement these could be reflected on when already in a negative mood (or not at all) so that positive moods are not impaired. We found that negative reflection when in a negative mood does not reduce current mood, and is associated with one aspect of well-being when reflections are past-focused.

However, our work suggests other important new implications that extend beyond simple mood adjustments. For example, we found that current mood affects how we *remember* past experiences, and in some cases can be associated with improvements (or reductions) in well-being. We found evidence of predicted mechanisms previously shown to influence well-being in prior mood-agnostic unmediated reflection contexts (Petrie et al. 1998; Campbell and Pennebaker 2003; Wildschut et al. 2006). For example, we found greater redemption and distancing for negative reflection when in a positive mood. Conversely, there was more kill-joy thinking for positive reflection in a negative mood. However, an important new result is our demonstration that each of these incongruent reflection strategies had a cost, whereby negativity posed a threat to positivity and specific well-being aspects. For example, the Incongruent Negative group experienced greater redemption and distancing, but this did not improve general well-being, and in fact negative memories that overcame one’s currently positive outlook were associated with reductions in one well-being scale. And although the Incongruent Positive group received a boost to their negative mood, this was at the cost of contaminating the positive memory, reducing one aspect of well-being if reflections were focused on this contaminated past. Thus while considering incongruent reflection as a strategy for mood-regulation, one must also consider possible consequences of negative moods and memories. Negative memories can reduce positive moods, and negative moods can contaminate positive memories. In other words, negativity can trigger kill-joy thinking in two ways: *Kill*-*joy memory*, and *kill*-*joy mood*. And both can detract from well-being.

While we predicted the Congruent Negative group would have trouble distancing themselves from memories (as compared to the Incongruent Negative group), we were surprised that they also experienced increases in one of our well-being scales when their reflections were past-focused. Why then might it be adaptive to focus our reflections on the negative past when in a negative mood, rather than seeking to put distance between where we were then, and where we are now? A phenomenon called *depression realism* (Alloy and Abramson 1979) might shed light on this finding, whereby those in a depressed mood are more likely to be accurate and realistic in their inferences. While the theory is not without criticism (Benassi and Mahler 1985; Dunning and Story 1991), support for it has been found in multiple contexts (Alloy and Abramson 1979; Keller et al. 2002; Seidel et al. 2012). Because negative memories can represent a problem to be solved (Bohanek et al. 2005), it’s possible that solutions are more accessible when a negative mood can provide an accurate outlook, contributing to well-being. In contrast, a positive mood might provide a positively-biased perspective, either interfering with problem-solving, or making revisiting the negative past more challenging. There may be resistance to taking an honest inventory of the past to identify solutions if this is at the expense of reducing one’s currently positive mood. A negative memory might be easier to work through if already in a negative mood where there is nothing left to lose.

There is an alternative explanation for why focusing on past negative memories when in a negative mood was associated with increases in one of our well-being scales. It is possible that these memories served as distracters from current situations that were causing a negative mood. When the negative memory was effective at taking one’s mind off the present moment (as indicated by past tense words in reflections) this was associated with one aspect of well-being. However, when one was swept up in the drama of the moment, the negative memory failed as a distracter. The latter scenario was described in an exit interview by a participant who said negative memories “would come when I was in a bad mood so I was more focused on my present bad mood than my past.” Interestingly, while positive memories might be thought of as exemplary distracters for negative moods (Nolen-Hoeksema 1991), this group showed reductions in one of our well-being scales when focused on the past. Thus the kill-joy aspects of the Incongruent Positive group seemed to outweigh potential distracter benefits.

Also, unexpectedly, we did not find group differences or overall changes in well-being. And yet we did find evidence of redemption and distancing, two mechanisms shown in prior unmediated research to drive well-being and general health improvements (Campbell and Pennebaker 2003; Wildschut et al. 2006). Additionally, Isaacs et al. (2013) employed a similar methodology and demonstrated the well-being benefits of TMR. Why then did we not find these changes in the current study? There are at least two possibilities stemming from methodological differences.

First, the prior TMR study had participants reflect more frequently, yielding an average of 53.42 reflections each (Isaacs et al. 2013) compared with 9.43 times in the current study. Our decision to reduce the number of reflections was motivated by our pilot study which showed people were comfortable writing up to 15 memories of each valence. Finding no overall well-being changes in our current study might result from participants experiencing fewer reflections compared with prior work.

Our second methodological choice was for participants to generate a pool of memories at the start of the study, with some memories being selected later for reflection. In the prior study, participants recorded experiences as they occurred over the course of the study. This concurrent strategy yields a larger pool of memories that tend to be less emotional, since the window of time to capture emotional experiences is limited to the study length. For the current study, we needed memories that were clearly emotional, as mildly emotional memories might not evoke measurable effects. Thus, generating a memory pool at the start of the current study was preferable, allowing participants to self-select highly emotional memories from their recent past. This choice was supported by our finding of redemption and distancing in reflections, indicating that memories were emotional and recent enough to undergo these changes. One reason why we did not find evidence of understanding (words that indicate insight and cognitive processing) is that the writing of memories for the pool might have elicited increased understanding *before* the intervention. Thus, not finding well-being changes in our study could have been due to less exposure to memories, as well as memories that might not have been open enough for learning opportunities.

Our findings suggest many new opportunities for future research. For example, a longer-term study could have participants record many experiences as they happen over a couple of months. This would generate a larger pool of memories that are recent so that participants are exposed to a greater number of reflections. Such a study would help resolve observed inconsistencies in well-being mechanisms without well-being results, though at the risk of higher participant dropout due to an extended study length. Furthermore, while we used LIWC to investigate explicit hypotheses motivated by prior work, future research could take a more open and exploratory approach to LIWC that might yield new interesting mechanisms.

Our work also suggests multiple opportunities for redesigning current TMR systems to harness the influence of mood. For example, systems could strategically select specific types of memories to be reflected on when users are in particular moods. So, if the goal of Facebook’s On This Day is to provide users with a small amount of enjoyment and upliftment, positive memories would likely be more effective when the user is in a negative mood. Current mood might be assessed by providing lightweight mood probes (as in our study) or algorithmically by analyzing affect words used in various online behaviors (Desmet 2005; Kramer et al. 2014). However, there may be a cost of kill-joy memory and reduced well-being if the user’s reflection is past-focused. Now, when a user shares an On This Day memory, they are prompted to “Say something about this…” but this could be restructured to prompt a style of reflection that facilitates benefits while circumventing costs. For example, On This Day might prompt the user to think about how the memory applies to their life either currently or in the future, to help reduce devolvement into past-focused contamination. Additionally, just as On This Day includes a rich diversity of media types, mood and memory interactions could also be explored for images, videos, or even music in future work.

If a TMR system resurfaces negative memories, users might receive a boost to their well-being if they are already in a negative mood and the system prompts reflections written in the past tense. When in a positive mood, if the system detects negative emotion words as the user reflects, it might intervene by encouraging greater distancing and redemption from negative memories so as to resist kill-joy mood, and preserve well-being. This might be accomplished by encouraging writing in third person (for distancing) or prompting positive reappraisal (for redemption). Future research could uncover whether such structured prompts have the desired impact and how to do this subtly so as not to undermine the user’s experience.

However, systems that select memories for negative moods shouldn’t rely on this feature centrally. MoodAdaptor selected memories for negative moods more actively than positive moods, but the total frequency of such reflections was still low due to the sparsity of people’s negative moods. Designing for negative moods is a challenge for normal populations, although our study provides some insights for which memories to select when negative moods do occasionally occur. Additionally, there may be extreme cases where negative memories require a realistic outlook only accessible in a negative mood state for them to be fully processed. As with all mood-adapting TMR systems, short-term mood adjustments need to be carefully weighed against more general well-being effects.

## Conclusion

Autobiographical memory enhances our positivity through well-documented self-enhancement biases. Our current mood also influences our memories by selecting experiences that are emotionally congruent (and sometimes incongruent) with our current mood. However, prior work has not addressed fundamental questions about how mood might influence written reflection, and in turn how such reflection might influence mood. By designing MoodAdaptor and deploying it in a real-world setting, we were able to answer theoretically-motivated questions about the relationship between mood, reflection and well-being when using technology. A systematic analysis of the words people used in their memories and reflections uncovered the mechanisms behind these relationships. Our findings reveal a competition between positivity and negativity in our moods and memories, yielding adaptive mechanisms when positivity prevails, and contamination when negativity overshadows. This extends theories of well-being and opens up exciting new opportunities for future research. Lastly, our findings provide new insights about how to design impactful TMR systems that harness mood.
